# How much self-poisoning attempts are visible in Iran?

**DOI:** 10.5249/jivr.v10i2.914

**Published:** 2018-07

**Authors:** Mehdi Moradinazar, Farid Najafi, Mohammad Reza Baneshi, Ali Akbar Haghdoost

**Affiliations:** ^*a*^Modeling in Health Research Center, Institute for Futures Studies in Health, Kerman University of Medical Sciences, Kerman, Iran.; ^*b*^Research Center for Environmental Determinants of Health (ECEDH), School of Public Health, Kermanshah University of Medical Sciences, Kermanshah, Iran.

**Keywords:** Visibility, Self-poisoning, Suicide attempts (SA)

## Abstract

**Background:**

Stigma of suicide attempt (SA) results in not asking friends and relatives for help. Others’ awareness of an individual’s SA sometimes can solve his/her problems and reduce rates of SA. This study is intended to examine the degree of SA visibility through deliberate self-poisoning (DSP), which is the most common method of SA in Iran.

**Methods:**

In order to study visibility, all individuals who had attempted to suicide by DSP and had been referred to the western Iran poisoning center during April-June, 2016 were entered to the study. A female and a male interviewer experienced in role-playing were recruited to interview clients, each with clients of their own gender, in order to increase compliance and information accuracy. Multivariate Poisson Regression was used to identify visibility determinants.

**Results:**

Among 100 subjects interviewed, 10 denied SA. Regardless of those denying SA, self-poisoning visibility factor (SVF) was 26.6% (21.7-31.5) which decreased to 23.9% (19.7-28.1) after considering those individuals who denied SA. The highest values of SVF were observed in subjects poisoned by toxins, alcohol and illegal drugs, respectively. In the multivariate model, the value of SVF increased with an increase in age (IRR=1.03, 95% CI: 1.02-1.04), having history of SA (IRR=1.18, 95% CI: 1.07-1.30), and being married (IRR=1.70, 95% CI: 1.05-1.29).

**Conclusions:**

Lower values of SVF of DSP indicate that individuals committing suicide do not ask others for help and saying their SA intents. The higher the degree of visibility, the lower the rates of committing and repeating SAs. To increase the visibility of SA, therefore, the one way is to prevent and reduce SA repetition.

## Introduction

Worrisome growth of SA rates in recent decades and its social, economic and psychological consequences have prompted World Health Organization (WHO) to introduce reduction of SA rates around the world as one of its objectives within a public health program.^1,2^ Like other countries, Iran has seen a considerable increase in SA rates in recent decades; therefore, it is among issues of interest into the realm of mental health, especially in teenage and youth age groups.^3^ Most societies consider suicide as a stigma, causing the person who committing suicide not to ask others for help. As a result, thinking of and committing suicide are mostly hidden from the sight of relatives and friends,^4,5^ who become aware of this fact too late when there is a bitter, unpleasant memory left. When people surrounding become aware of a person’s decision to commit suicide, they can help with solving his or her problems and reduce SA rates.^6^ In traditional societies like Iran, the social stigma of SA is probably higher in comparison with developed ones. ^7^ SA denouncement by society leads to not disclosing such an attempt. Hiding and lack of reporting on suicide results in under-counting and under-reporting of SAs.^8^

Given the first step toward planning for the long-term is to acquire correct and precise numbers and information, so failure of preventive actions and planning to reduce SA rates are anticipated in most cases. Recently, various methods have been developed to estimate the number of hidden populations like those committing suicide. One of the simplest and most commonly used of which is network scale up (NSU) method. This method is based on an inventory study of a random sample from the general population,^9^ which gives an estimation of the size of the target group by using information of respondent’s personal networks. However, since this method assumes that respondents know all information about people belonging to their own personal networks, it may underestimate the size of groups hiding their behaviors. This problem is termed transmission error,^10^ which, in fact, occurs when respondents are not aware of behaviors of an individual belonging to their own personal network.^9,11^ In order to remove transmission error from estimated size of a target group, we should calculate its social visibility, that is, we should determine what percentage of society members are aware of the group’s behaviors at first and correct it's estimated size next.^12^

Knowing social visibility of SA not only is applied to NSU-based correction method to estimate the number of SA cases, but also can be an approximate estimate for the degree of unacceptability and non-disclosure of SA. In Iran, DSP is the most commonly used method of SA,^7^ accounting for 2/3 of SA cases and more than 1/3 of SAs leading to death.^13^ During recent years, the number of DSP cases has grown more increasingly than that of other SA methods. ^14^ Therefore, the present study examines the degree of visibility and the factors influencing it regarding individuals committing suicide by DSP as the most common method of SA.

## Methods 

**Study population**

This cross-sectional study was carried out in Imam Khomeini Treatment Center, Kermanshah, Western Iran. Having about two-million populations, Kermanshah is the most populated western province in Iran. Also, Kermanshah province shares common borders with Iraq. Imam Khomeini Treatment Center is the biggest, most equipped and principal referral poisoning and burns treatment center in the western part of Iran as a treatment pole. More than 90% of (non-) deliberate self-poisoning cases as well as nearly 100% of burning cases needing treatment are referred to this center. In order to examine visibility, all individuals who had committed suicide by DSP during April-June of 2016 and referred to the center in the morning and have lived in Kermanshah in recent five years were entered the study.

**Data collection and measurements**

Interviews were performed in a quiet, private room, where patients can feel relaxed and secure. After stability of patients conditions, the interviews were done in order to estimate visibility of SA. To increase compliance and information accuracy, a female and a male interviewer trained about role playing were recruited to ask related questions from clients of their own gender. Clients were informed of the research goals prior to perform it. After that, they were assured that collected data would be kept confidential and used to do a research work. Data were collected without recording the clients’ names in order to observe ethical principles. In addition, the present research was done based on all principles and rules of confidentiality. All stages of the study design and implementation were approved by the Ethical committee of Kerman University of Medical Sciences (IR. KMU.REC. 2015. 440).

A questionnaire was developed to perform interviews accordingly, the first part of which explained the study objectives, and the second determined the number of relatives, friends, neighbors, and coworkers of individuals committing suicide, who knew them. That is, respondents knew them by their names and/or faces, met them at least once during 2 past years, and/or had telephone or e-mail contacts with them. Those over 18, among the respondents who knew clients in this way, were selected and classified into 3 groups as follows: probably being aware of SA, surely being aware of SA, and surely not being aware of SA. In order to increase the precision of the study, the number of relatives, friends, neighbors, and coworkers was determined separately, that is, if a person fitted in 2 or more groups, he/she would be counted only once in one group. For example, if a person was a friend, a coworker and a relative of a client, the former would be counted only by one group. In the third part of the questionnaire, in addition to demographic information about people committing suicide, the number of SAs, types of toxic substance, and the number of people who knew individual (client) concerned who committed or died from suicide were also determined.

**Data management and statistical methods**

After collecting data, the rates of SA visibility were calculated by following formula:

$$ Self-poisoning Visibility Factor= \frac{The number of people who were surely aware of SA}{Total number of familiar people} $$

Confidence interval (CI) was determined at 95% by using bootstrap method with 1000 repeats.

We used Poisson regression when the dependent variable was numerical and had a Poisson distribution. In Poisson regression model, the exponents of coefficients are equal to the incidence rate ratio (IRR), which is a synonym for relative risk.

Any variable having P<0.3 in univariate model was entered into multivariate model at which all variables with P>0.05 were excluded from final model by using backward method and merely variables with P<0.05 were included in the final model. Data were analyzed using STATA software (Version 14.1; Stat Corp, Texas, USA).

**Ethical statement**

In order to collect information based on NSU method, we did not record the name of interviewees. All stages of the study design and implementation were approved by Ethical Committee of Kerman University of Medical Sciences (IR. KMU.REC. 2015. 440).

## Results

Twenty four (54%) and 46 (46%) Of 100 individuals committing suicide by DSP (X 60-90) during April-June of 2016 were women and men, respectively. Among these clients, 38 (38%) committed DSP by taking medications, 27 (27%) by poisons and agricultural toxic substances, 20 (20%) by using alcohol and illegal drugs, and 15 (15%) by using Kerosene and detergents. Ten out of 100 clients committing suicide denied SA. Fourteen (15.5%) out of remaining clients, who did not deny SA, had a history of previous SAs. Clients committing suicide were familiar, on the average, with 1.07±1.3 individuals committing suicide of whom 0.69±0.8, on the average, committed non-fatal suicide (Table 1).

**Table 1:** Distribution of SA through DSP and the number of suicides known, 2016

Variable		Frequency (%)	Number of Suicides known		
			Total Mean (SD)	Non-Fatal Mean (SD)	Fatal Mean(SD)
**Gender**	Male	40(44.5)	1.1(1.3)	0.7(0.8)	0.4 (0.6)
	Female	50(55.5)	1.0(1.3)	0.7(0.7)	0.4(0.6)
**Age group**	≤20 years	31(34.4)	0.7(1.0)	0.6(0.7)	0.2(0.4)
	21-30 years	40(44.4)	1.2(1.3)	0.7(0.8)	0.5(0.7)
	≥31 years	19(21.2)	1.3(1.6)	0.8(0.9)	0.5(0.8)
**Marital status**	Single	58(64.4)	1.2(1.4)	0.8(0.8)	0.3(0.7)
	Married	32(35.6)	0.9(1.2)	0.5(0.7)	0.4(0.6)
	≤5	9(10.0)	1.9(1.1)	1.1(0.8)	0.7(0.4)
**Level of education (year)**	6-9	26(28.9)	1.2(1.3)	0.7(0.8)	0.5(0.7)
	10-12	38(42.2)	0.9(1.3)	0.6(0.8)	0.3(0.7)
	≥13	17(18.9)	0.7(1.3)	0.5(0.9)	0.1(0.7)
**Method of poisoning**	Drug	30(33.3)	1.0(1.2)	0.7(0.8)	0.3(0.6)
	Poison	25(27.8)	1.5(1.8)	0.9(1.0)	0.7(0.8)
	Narcotics & alcohol	20(22.2)	0.9(1.0)	0.6 (0.6)	0.3(0.4)
	Kerosene	15(16.7)	1.0(1.1)	0.7(0.6)	0.3(0.4)
**History of suicide**	No	76(84.4)	1.1(1.3)	0.7(0.8)	0.3(0.6)
	Yes	14(15.6)	1.1(1.4)	0.1(0.8)	0.3(07)
**Total**	****	**90(100)**	**1.07(1.3)**	**0.69(0.8)**	**0.39(0.6)**

Value of SVF for clients committing DSP was 26.6% (21.7-31.5) without taking SA deniers into account, which decreased to 23.9% (19.7-28.1) when taking SA deniers into account. As shown byFigure 1, different methods of DSP indicate significantly different SVF values (P<0.001).

**Figure 1:**
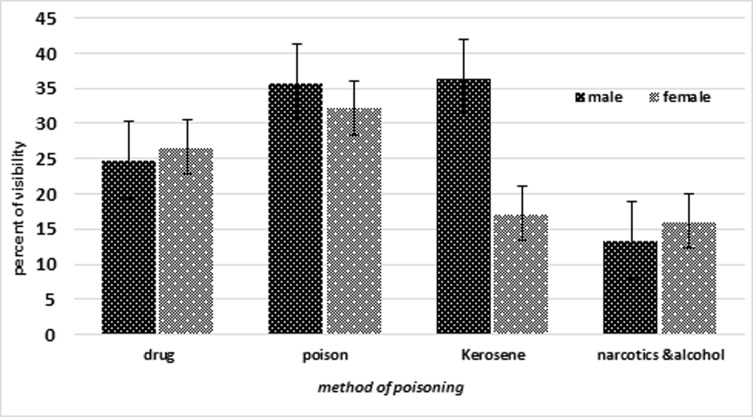
Percent of visibility by DSP methods in suicide attempted persons

The highest and lowest values of SVF were related to poisoning by toxic substances and by alcohol and illegal drugs, respectively, but there are no significantly different SVF values for both genders (P=0.7).

At both univariate and multivariate model without considering SA deniers, evaluation of factors influencing SVF indicated that SVF values increased as age increased so that SVF values were higher for clients over 30 than those for clients under 20 (Crude Incidence Rate Ratio (IRR)=1.53, 95% CI: 1.37-1.72; adjusted IRR= 1.87, 95% CI: 1.60-2.16).

In multivariate model, history of SA (IRR=1.18, 95% CI: 1.07-1.30) and marital status (IRR= 1.70, 95% CI: 1.05-1.29) had significant relationships with SVF after adjusting the potential determinants (Table 2).

**Table 2:** Determinants of SVF in univariate and multivariate poisson regression

Determinants		Crude IRR (95% CI)	Adjusted IRR (95% CI)	P Value
**Gender**	Male	1	---	---
	Female	0.99(0.91-1.07)	---	---
**Age group**	≤20 years	1	1	
	21-30 years	1.40(1.29-1.60)	1.57(1.40-1.77)	<0.001
	≥31 years	1.53(1.37-1.72)	1.87(1.60-2.16)	<0.001
**Marital status**	Single	1	1	
	Married	0.94(0.87-1.02)	1.70(1.05-2.29)	0.002
**Level of education (year)**	≤5	1	1	
	6-9	0.98(0.85-1.12)	1.20(1.03-1.40)	0.01
	10-12	1.13(1.0-1.28)	1.45(1.26-1.66)	0.001
	≥13	0.80(0.69-0.90)	0.90(0.77-1.04)	<0.001
**Method of poisoning**	Drug	1	1	
	Poison	1.27(1.15-1.39)	1.18(1.07-1.30)	0.001
	Narcotics & alcohol	0.90(0.75-1.07)	0.96(0.81-1.15)	<0.001
	Kerosene	0.7(0.61-0.79)	0.72(0.63-0.82)	<0.001
**History of suicide**	No	1	1	
	Yes	1.08(0.95-1.21)	1.18(1.07-1.30)	0.01

## Discussion

Feeling the needs for others’ help and sympathy to meet personal needs, for others’ support, and for others’ love and affection are among factors, when not met, cause some people to commit suicide, in fact, the need for others’ help is caused by the need for having a sympathetic, trustworthy, attentive and kind supporter. Most individuals committing suicide acknowledged that the reasons why they did so were to escape from situations they were in and to be unable to tolerate such conditions. In reality, most of such individuals do not intend to die, but rather they want people around them to become aware of their feelings and emotions.^7^

In the present study, more than 1/3 of DSPs were committed by using medications, which is in agreement with similar studies performed in Iran.^7^ In other countries, different substances are used to commit suicide depending on their own cultures and on the types of substances available. As is with Iran, for example, in western and European countries, medications are the most common and notable substances used to commit suicide.^15,16^ But in Southeast Asia, especially in Sri Lanka, Kerosene and agricultural toxins are used to commit DSP, respectively.^17,19^ In rural areas of Iran, poisons and chemicals are the most important toxic substances.^20^

Values of SVF increase with an increase in potency, in volume and in a number of toxic substances.

Given that poisons and chemicals are highly virulent, DSPs committed by these substances show maximum SVF values while minimum SVF values are shown when alcohol and illegal drugs are used. One reason for why SVF values are low for individuals poisoned by alcohol and illegal drugs is that it is unknown whether they used them to feel pleasure and enjoyment or to commit suicide. As a result, SAs are denied more easily.

In this study, nearly 15% of individuals committing suicide had a history of previous SAs; this figure was between 10% and 28% in other similar studies. This difference can be justified by the rates of SAs and of deaths from them in different societies. In general, rates of previous SA history are higher in areas where rates of death from SAs are high. Values of SVF were higher for people who committed suicide previously than those for people who did so for the first time. Earlier studies have shown that some individuals had used more potent substances with higher volumes when they committed repeated suicides; therefore, it cannot be argued that making use of more potent toxic substances is one possible reason why values of SVF are higher for people with a history of SAs. Also, with older people, SVF values increased at both univariate and multivariate model. There are 2 possible reasons for increased SVF values with increased ages: first, reduction of SA denials and not stating SAs had been committed accidentally; and second, making use by older people of more potent and risky toxic substances. Typically, older people have access to such substances more easily.

Psychologists and experts believe that DSP of SAs are more of malingering aspects, that is, individuals use medications and poisons in order to attract others’ attention and/or to achieve a particular goal. For this reason, rates of SA by poisoning were higher among women than men in this study as well as in similar studies,7 but no significant differences were observed in SVF values between women and men.

Less than 30% of friends and relatives of individuals committing suicide were aware of their SA intention, indicating the strength of SA stigma in society. Given low values of SVF, it can be said that suicidal thoughts and attempts are hidden from the sight of relatives and friends. If relatives become aware of SA intents, they will help individual committing suicide to solve their problems and reduce SA rates, especially in traditional societies like Iran where most problems are solved by relatives and other people around. Although such awareness can reduce the rate of SAs, it can also increase the likelihood of SAs by 65%. Such a phenomenon is referred to as transmission of suicide.^21,22^

Similar studies show that 30-60% of people committing suicide had not informed others of their intents.^23^

Given that similar studies have demonstrated that most of individuals committing suicide have reported no serious intents to kill themselves, informing and communing with others may reduce the rates of SAs and repeated SAs.^24^

**Strengths and weaknesses**

Present study had some limitations, the most notable of which included small sample size, lack of cooperation between subjects, and clients’ lowered consciousness levels caused by poisoning complications. In order to prevent bias, interviewers performed interviews after patients’ conditions had become stable. Another limitation of this study was denial of SAs by individuals committing suicide. To solve this problem, we calculated SVF values with and without considering such subjects. On the other hand, present study had several strengths including selection of all individuals committing suicide who were referred to the most important treatment center in the western Iran during a 3- month time period; recruitment of trained staffs, provision of a quiet place to perform interviews; and asking indirect questions (about social networks).

## Conclusions

Based on the findings of this study, SVF of SAs is extremely low. The more virulent the method of SA, the lower the SVF values. The highest and lowest values of SVF were observed with DSPs by using poisons and by using alcohol and illegal drugs, respectively. 

In sum, SVF is lower for individuals committing DSP because they do not ask for help. The higher the visibility of SAs, the lower the rates of SAs and of repeated SAs. For this reason, one way to prevent and reduce repeated SAs is to increase visibility of SAs.

**Acknowledgment**

Ali Akbar Haghdoost and Dr. Mehdi Moradinazar are involved in planning, implementation and data collection of the study and contributed in drafting the manuscript.

Dr. Farid Najafi and Mohammad Reza Baneshi are involved in planning, implementation and data collection of the study and contributed in drafting the manuscript. All authors have read and approved the final manuscript. 
